# Outcomes and Correlates of Major Depression in 11 Disaster Studies Using Consistent Methods

**DOI:** 10.3390/bs11010004

**Published:** 2021-01-01

**Authors:** Carol S. North, David Baron

**Affiliations:** 1 The Altshuler Center for Education & Research, Metrocare Services, Dallas, TX 75247, USA; Carol.North@UTSouthwestern.edu; 2 Department of Psychiatry, The University of Texas Southwestern Medical Center, Dallas, TX 75390-9070, USA; 3 Department of Psychiatry, Western University of Health Sciences, Pomona, CA 91766, USA

**Keywords:** postdisaster depression, disaster mental health, functional impairment

## Abstract

This study investigated psychosocial functioning and employment status in association with postdisaster major depression and its course in survivors of 11 different disasters in a sample of 808 directly-exposed survivors of 10 disasters and 373 survivors of the 11 September 2001 (9/11), terrorist attacks on New York City’s World Trade Center (total *n* = 1181). Participants were assessed between 1987 and 2007 with structured diagnostic interviews in a prospective longitudinal design. Consistent research methods allowed merging of the disaster databases for analysis using multivariate modeling. Postdisaster major depression in the study cohort from the 9/11 disaster was more than twice as prevalent as in the other disasters, possibly reflecting the greater psychosocial/interpersonal loss and bereavement experienced by 9/11 disaster survivors. At follow up, employment was associated with remission of postdisaster major depression, non-development of PTSD, and coping via family or friends. Functioning problems were associated with disaster injuries, but not with persistent major depression. This study is unprecedented in its large sample of survivors across the full range of disaster typology studied using consistent methods and full structured interview diagnostic assessment. These findings may help guide future interventions to address postdisaster depression.

## 1. Introduction

Major depressive disorder (MDD) is the mental disorder causing the most functional disability, and it represents a major source of psychosocial impairment globally [1,2,3,4,5]. However, the place of MDD in disaster mental health research has been eclipsed by the centrality of posttraumatic stress disorder (PTSD) in this research [6,7,8,9,10,11]. Disaster mental health research has established the association of considerable functional disability with PTSD [12,13,14,15,16,17] and identified risk factors for PTSD development [6,18,19] but not predictors of PTSD remission [20,21,22].

Comparatively little is known about the temporal course of MDD, predictors of MDD remission, and functional outcomes of MDD in disaster settings [17]. A few studies have found that MDD, like PTSD, is associated with functional impairment [16,17,22,23]. A study of directly exposed survivors of a terrorist bombing in Kenya found a higher number of functioning problems in association with MDD than with PTSD [22]. Another study, of young adult survivors of the sinking of a cruise ship, found greater evidence of functional impairment in association with MDD than with PTSD [16]. That study also found specificity of impairment of types of functioning with type of disorder: love relationships and vocational functioning for MDD and friendships and nonspecific social relationships for PTSD [16]. Studies have shown that recovery from posttraumatic psychopathology was not associated functional outcomes [16,24] and that the improvement in functioning was not reflective of the amount of recovery from psychopathology [23], but little is known about improvement of functioning in relation to recovery from MDD specifically.

Clearly, research on MDD has lagged behind PTSD research in disaster settings, particularly with respect to examining associations of MDD with impaired functioning and predictors of remission of the disorder. This article presents an analysis of outcomes and correlates of postdisaster major depression—specifically, prediction of remission from the disorder and association of the disorder and remission from it with psychosocial functioning, including employment status—in a prospective longitudinal study of total of 1181 survivors of 11 different disasters.

## 2. Methods

The merging of data into two main databases for comparison and replication of findings across them was made possible because of consistent use of research methods across all 11 disaster studies, including initiation of data collection as soon as feasible after the first postdisaster month, use of representative and systematic sampling methods when possible through universal and random sampling from lists of employees of affected businesses or official registries and geographic maps of disaster survivors and damaged households, and structured interviews collecting the same variables across all studies assessing disaster-related experiences and psychiatric disorders with onset and recency information keyed to the date of the disaster [25]. The first database includes data from studies of 808 directly-exposed survivors of 10 disasters that occurred between 1987 and 1995, which represented all types of disaster: natural disasters (tornado, floods, earthquake), technological accidents (plane crash into hotel, firestorm), and intentionally-caused disasters (four mass murder episodes and a terrorist bombing) studied at a median of three months (average range, 1–6 months) post disaster. Part of this 10-disaster sample was obtained through systematic recruitment (six disasters; participation rate = 77% of *n* = 683) The remainder (four disasters, *n* = 282) was recruited through letters mailed to homes in the disaster zone or distributed by affected workplace management to employees, with unknown participation rates, because there was no way to trace how many survivors who had moved from the disaster-ravaged communities received the letters or how extensively the letters were distributed to employees, and thus these samples were assumed to represent volunteer samples. Follow-up interviews were conducted at one or three years or both. Follow-up variables included in these analyses represent data collected at the last follow-up interview (one year for *n* = 647 and three years for *n* = 69). The overall follow-up rate was 89% (of *n* = 808). A seven-year follow-up study was conducted only for the Oklahoma City bombing sample [24]; these data were not included in the one- and three-year follow-up 10-disaster database but were used for separate analysis of baseline and seven-year follow-up functioning variables, which were not collected for the other nine disasters in this database.

The second database represents a volunteer sample of 373 survivors of the 11 September 2001 (9/11), terrorist attacks on New York City’s (NYC) World Trade Center (WTC) who were recruited from employees of eight NYC organizations affected by the attacks. The sample included 171 employees of businesses in the WTC towers and 202 others employed elsewhere in the geographic vicinity, and 27% of the entire sample of *n* = 373 had direct exposure to 9/11 disaster trauma. Baseline interviews were conducted at a mean of 35 months after the disaster. A follow-up study was conducted approximately six years after the disaster. The 9/11 follow-up participation rate was 61% (of *n* = 373) which included 73% of WTC tower employees.

Space limitations preclude the presentation of the full methodological details, but extensive information regarding the sampling methods, collection of research data, and characteristics of the samples is available in previous publications [19,24,26,27,28]. Institutional Review Board (IRB) approval for human subject research was obtained in advance, and all participants provided written informed consent at the time of enrollment into the study.

Psychiatric diagnoses were assessed with the Diagnostic Interview Schedule (DIS) [29]. Onset and recency questions were specifically queried in relation to the date of the disaster, permitting diagnosis of lifetime predisaster, postdisaster, and incident disorders after the disaster, as well as timing of remission of disorders. Demographic information was collected on sex, age, race/ethnicity, marital status, and educational attainment. The Disaster Supplement [30] provided information on participants’ disaster experience. It was tailored to the specific characteristics of each disaster, and variables collected in common to all of the disasters included exposure to disaster trauma according to diagnostic criteria for PTSD (directly exposed, witnessed exposures, and indirect exposures to close associates who experienced disaster trauma) and personal injury in the disaster. The Disaster Supplement also asked participants how they coped with their feelings of upset about the disaster in four categories (through family and/or friends, seeking mental health treatment, drinking alcohol, and taking medication).

Functioning variables were collected from 75 Oklahoma City bombing study participants at baseline and at seven-year follow up and from 158 9/11 study participants at baseline and six-year follow up, but not from other disaster sites included in this study. The functioning information was obtained through 27 “Yes/No” items assessing a variety of problems in functioning in response to exposure to the disaster at baseline and in the last year at follow up, in four partially overlapping domains: home (difficulties with participating in home activities, following household routines, completing chores, and interacting with family members), work (difficulties with participating in work activities, completing work, understanding tasks, concentrating, interacting with supervisors and coworkers, and following work policy), social/recreational (difficulties with participating in social and recreational activities, interacting with friends, relaxing, and enjoying social/recreational activities), and personal/interpersonal domains (difficulties with interacting, getting along, and cooperating with others; with ability to relax and enjoy; and with emotional upset in home, work, and social/recreational settings) in the current year and in the first disaster weeks and months, collected retrospectively in the Oklahoma City dataset and prospectively (baseline and follow-up) in the 9/11 dataset. Simple counts of positively endorsed functioning items were made.

The original version of the DIS used for the 10-disaster data collection queried only symptoms of the worst lifetime depressive episode, and therefore postdisaster depressive symptoms in the 10-disaster dataset represent symptoms of only the identified worst lifetime depressive episode, with those not describing a worst episode as occurring after the disaster being counted as having no postdisaster depressive symptoms. Modification of the DIS for the 9/11 study permitted specific inquiry about symptoms of all (not just those of the worst) postdisaster depressive episodes.

### Data Analysis

The 9/11 WTC and 10-disaster databases were combined into a single database representing all 11 disasters (*n* = 1181 at baseline, 942 at follow up) for comparison and/or replication of relevant 10-disaster database analysis with 9/11 database analysis. The data for the 10-disaster database were entered into separate Microsoft Access datasets for each disaster, reflecting the same variables from identical interviews collected across all 10 disasters. The data from the two disaster databases were then combined into a single database using the “DATA…SET” command in SAS. For comparison of the 10-disaster data with the 9/11 data, these two databases were combined using a similar procedure, as the variables to be compared and/or replicated in these two databases were collected with the same interview questions.

Data analysis was conducted using SAS 9.4 (SAS Institute, Cary, NC, USA). Chi-square tests were used to compare two dichotomous variables (substituting Fisher exact tests for expected cell sizes <5). Paired t-tests were used to compare numerical variables measured in the same individuals at different time points.

Multivariate logistic regression models with survivors nested within disasters (using PROC GLIMMIX in SAS) were used to predict remission of any postdisaster major depression, employment, and functioning at follow up (dependent variables, one per model). Independent covariates entered simultaneously into all of these models included baseline demographics (sex, age, racial minority, married status, college education), trauma exposure variables (injury in the disaster and also direct exposure, witnessed exposure, and indirect exposure for the 9/11 data), baseline diagnostic variables (predisaster major depression disaster-related PTSD, postdisaster alcohol abuse/dependence), and baseline methods of coping with upset about the disaster (through family/friends, mental health treatment, drinking alcohol, and use of medication).

For prediction of remission of any postdisaster major depression, only survivors who were diagnosed at baseline with postdisaster major depression were included (models presented in Table 1). For prediction of current employment at follow up, only survivors who were employed at the time of the disaster were included, with baseline postdisaster major depression added as an independent covariate; separate models were tested that included only survivors with baseline postdisaster major depression and addition of remission of any postdisaster major depression as an independent covariate (models presented in Table 2). Prediction of functioning at follow up was conducted only with the Oklahoma City bombing data and 9/11 data, which included the functioning variables. Functioning at follow up was predicted from baseline postdisaster major depression in the entire sample; separate models were tested that included only survivors with baseline postdisaster major depression and addition of remission of any postdisaster major depression as an independent covariate (models presented in Table 3).

Variance inflation testing using the/VIF function of PROC REG was conducted to ensure an acceptable level of multicollinearity (generally < 1.8) among the independent variables included in the models. Statistical significance was set at α = 0.05.

## 3. Results

### 3.1. Postdisaster Major Depression Remission

Compared to the follow-up participants, the nonparticipants had a higher proportion with postdisaster major depression at the baseline interview (22% vs. 18%, *p* = 0.013). Follow-up participants and nonparticipants did not differ in proportions with baseline postdisaster or lifetime predisaster major depression.

In the 10-disaster database, postdisaster major depression was identified in 13% (of *n* = 714) at baseline, and in 22% (of *n* = 714) at any time after the disaster (baseline or follow up). At last interview, 66% (of *n* = 156) of those with any postdisaster major depression were in remission. Postdisaster major depression and its remission did not differ by timing of last follow up (one vs. three years). The lifetime predisaster prevalence of major depression was 14% (of *n* = 714). In multivariate analysis of the 10-disaster database among the subset with any postdisaster major depression, remission from postdisaster major depression at 1–3 year follow up was positively associated with married status and negatively associated with coping with the disaster through use of medication (see Table 1).

In the 9/11 database, postdisaster major depression was identified in 32% (of *n* = 226) at baseline and at in 35% (of *n* = 228) any time after the disaster (baseline or follow up). At the six-year follow up, 86% (68/79) of those with any postdisaster major depression were in remission. The lifetime predisaster prevalence of major depression was 32% (of *n* = 228). Compared to findings in the 10-disaster database, the cumulative postdisaster major depression rate at any time in the 9/11 database was significantly higher (*p* < 0.001), and the remission rate was significantly higher (*p* = 0.001). The lifetime predisaster prevalence of major depression was also significantly higher in the 9/11 database (*p* < 0.001). In multivariate analysis of the 9/11 database among the subset with any postdisaster major depression, remission from postdisaster major depression at six years was negatively associated only with coping with the disaster through use of medication (see Table 1).

### 3.2. Employment

In the 10-disaster database, 77% (of *n* = 714) of all participants were employed at the time of the disaster; at the last follow-up assessment at one or three years, 71% (of *n* = 707) of the sample were currently employed. The vast majority (89% of *n* = 544) of survivors who were employed at baseline were currently working at last follow up; additionally, 14% (of *n* = 163) of people not employed at the time of the disaster were employed at last follow up. In multivariate analysis of the 10-disaster database among those employed at the time of the disaster, current employment at 1–3 year follow up was associated with younger age represented by a numeric variable of actual years of age but was not associated with baseline postdisaster major depression (see Table 2). Even among survivors younger than age 60 in separate analyses, the association still held (*p* = 0.011 in the 10-disaster database and *p* = 0.062 in the 9/11 database, not shown in tables). In a separate multivariate model of those employed at the time of the disaster who had any postdisaster major depression, current employment at follow up was not associated with remission from postdisaster major depression or with any other variable in the model (model not shown in tables).

In the 9/11 database, all of the participants were employed at the time of the disaster, consistent with the sampling from places of employment; at follow-up, 86% (of *n* = 226) were currently employed. In multivariate analysis of the 9/11 dataset, current employment at follow up was positively associated with younger age and was not associated with baseline postdisaster major depression (see Table 2). In a separate multivariate model including only the subset with any postdisaster major depression (also in Table 2), current employment at six years was positively associated with younger age, disaster-related PTSD, remission from postdisaster major depression, and coping through family and friends and negatively associated indirect exposure to disaster trauma.

### 3.3. Functioning Problems

Functioning data were available only in the Oklahoma City bombing site in the 10-disaster database and in the 9/11 data. Cronbach standardized coefficient α for the 27-item functioning list was 0.97 at index and 0.93 at follow up in the 10-disaster sample and 0.95 at index and 0.95 at follow up in the 9/11 sample, indicating excellent internal reliability.

In the Oklahoma City bombing data, the mean number of functioning problems at baseline was 11.2 (SD = 9.8) and at seven years was 1.7 (SD = 3.7), a significant reduction (*p* < 0.001) over time. In multivariate analysis of the Oklahoma City dataset, the number of functioning problems at seven years was significantly associated with injury in the bombing and with baseline postdisaster alcohol abuse/dependence (see Table 3), but the association with baseline postdisaster major depression fell just short of significance (*p* = 0.053; not listed in table). In a separate multivariate model including only the subset with any postdisaster major depression, the number of functioning problems at seven years was not associated with postdisaster major depression remission or with any other variable in the model (model not shown in tables).

In the 9/11 data, the mean number of functioning problems at baseline was 7.9 (SD = 7.7) and at six years was 1.4 (SD = 3.8), a significant reduction (*p* < 0.001) over time. In multivariate analysis of the 9/11 database, the number of current functioning problems at six years was not predicted by any postdisaster major depression or any other variable in the model (model not shown in tables). In a separate multivariate model including only the subset with any postdisaster major depression, the number of current functioning problems at six years was not associated with remission from postdisaster major depression or with any other variable in the model (model not shown in tables).

## 4. Discussion

This study examined outcomes and correlates of postdisaster major depression across two databases of a total of 1181 survivors from 11 disasters with collection of full diagnostic data. This analysis and comparison across disasters was made possible by the use of consistent methods across all 11 disaster studies.

The postdisaster prevalence of major depression in the 9/11 dataset was more than twice the postdisaster prevalence of major depression in the 10-disaster database. It has been suggested that these higher rates in the 9/11 disaster reflected the greater psychosocial/interpersonal loss and bereavement experienced by the 9/11 disaster survivors [9,28]. Remission of postdisaster major depression was associated with being currently married in the 10-disaster database, and it is intuitive that survivors with the support of a spouse might more readily overcome postdisaster depressive illness. In both databases, coping with the disaster through use of medication was associated with non-remission of postdisaster major depression. This likely does not mean that medication worsened the depression, but that people who used medication did so because they had more severe depressive illness to begin with.

Continued employment at follow up was associated with younger age in both the 10-disaster and 9/11 databases, which probably did not reflect attrition to retirement over time, because the relationship held in the subset of survivors under age 60. Employment at follow up was also associated with remission of postdisaster major depression and absence of disaster-related PTSD in the 9/11 dataset, which may represent an intuitive consequence of mental health on functioning at work. Additionally, the association of employment at follow up with coping through family or friends in the 9/11 dataset may reflect tangible effects of social support at the level of job functioning. Finally, the negative association of indirect exposure through close family or friends on employment at follow up in the 9/11 dataset, independent of recovery from major depression, may reflect added burdens on ability to function at the workplace resulting from shared suffering of the disaster exposures of loved ones.

In the Oklahoma City bombing data only, functioning problems were associated with injury in the disaster, but this was not replicated in the 9/11 dataset. It is understandable that physical injuries could have deleterious effects on functioning in the workplace. In the Oklahoma City bombing data only, postdisaster alcohol abuse/dependence, but not disaster-related PTSD or postdisaster major depression, was independently associated functioning problems. Because alcohol use disorder is not a regular mental health consequence of disasters [17,18,21,26,31,32], this association is likely not a product of exposure to the bombing. The lack of association between remission from major depression and functioning is similar to a prior finding that although disaster-related PTSD was associated with functioning problems at baseline in the Oklahoma City bombing study, by seven years, functioning problems were not associated with baseline PTSD or with recovery from it, suggesting that despite the effects of PTSD, these survivors managed to cope with the symptoms and return to functioning, even if their PTSD did not remit [33].

Variables not measured in the current study that could have further contributed to the mental health outcomes might include preexisting biopsychosocial risk factors, such as genetic loading for depressive disorders, other life stressors preceding the disasters but with clinical effects not emerging until after the disaster, and pre-existing coping strategies that were effective until the overwhelming emotional stress of the disaster exposure occurred. In the 9/11 database, the extensive and longstanding media coverage of this disaster was unprecedented, far more extensive than the other 10 disasters studied, and may have further contributed to the negative psychiatric consequences. Individuals who have engaged in psychotherapy may have learned techniques, such as cognitive restructuring, confronting automatic negative thoughts, and effective use of family and social supports during supportive psychotherapy to attenuate the emotional stress of their disaster trauma exposure. For MDD arising in the context of stressors, psychotherapy may play an especially important role in the recovery process relative to pharmacotherapy, which is likely the case in recovery from exposure to disaster trauma. The positive relationship of social support with continued employment in the current study is consistent with the robust associations previously found between social support and positive psychosocial outcomes in prior studies by this team and in the disaster literature in general, supporting the concept of the healing power of being surrounded by the support of trusted loved ones in the postdisaster workplace as well as at home.

This study’s strengths include a large (*n* = 1181) database of survivors of all major disaster types created by merging of samples from different disasters that was possible because of the use of consistent methods across all the studies. The nesting of the survivors within 10 different disasters in the analysis and comparing across disaster databases accounted for variability across disasters. Another strength of this study was the use of structured diagnostic interviews to assess full diagnostic criteria for psychiatric disorders in relation to the timing of the disasters. Study limitations include volunteer sampling introducing the possibility of sampling bias in some of the disaster sites, and potential recall bias based on length of time to data collection, especially in the 9/11 database.

These findings might suggest several implications for disaster mental health response. An obvious interpretation is that, as found in this study, that disasters that involve greater psychosocial and interpersonal loss can be expected to generate much more depressive illness, requiring more resources for mental health services to address this greater need. The finding that remission from postdisaster major depression was associated with being currently married suggests that disaster survivors without intimate partners might have more difficulty healing from major depressive episodes arising in the aftermath of disaster and may benefit from more intensive interpersonal emotional support as well as closer monitoring and management of their postdisaster course of psychiatric illness. The finding that remission of postdisaster major depressive illness was associated with employment at follow up is consistent with other research showing that extended isolation from usual daily routines such as work and school is associated with longer time to recovery and greater dysfunction.

If the association between productive activity and more favorable course of recovery is found to be causal, then it would support efforts to re-engage people with their workplaces and other systems of support to help stimulate recovery and return to normal functioning. Further, the association of employment with coping through social relationships suggests that social support may be fostered at the workplace or that social engagement may help retain people in the workforce—either way, this association is indicative of healthy associations between work and social relationships that can be fostered through progressive workplace programs or policies in the postdisaster setting. The finding that indirect disaster exposure through close associates was associated with not being employed at follow up suggests that such individuals may need additional support to help retain them in the workforce. Finally, the finding that non-remission from major depression was not associated with level of functioning suggests that patients can be reassured that even before their depression remits, they may be able to count on resumption of functioning, despite the fact that they are not feeling well yet.

This study has advanced existing knowledge about the presence and course of postdisaster major depressive disorder, because it was able to include a large sample by combining samples from several disasters through the use of consistent research methods and with diagnostic interviews that assessed full diagnostic criteria for major depression rather than using self-report symptom questionnaires or symptom screening tools. Additional studies of postdisaster major depression will need to apply rigorous methodologies including full diagnostic assessment and inclusion of many relevant variables in the analysis to clarify associations emerging from the data.

## Figures and Tables

**Table 1:** Multivariate models predicting postdisaster major depression remission in the 10-disaster and 9/11 databases ^a^.

	*p*	OR	95% CL
**10-disaster database**			
*Survivors with postdisaster major depression at baseline*			
Married status	0.046	3.46	0.63, 19.12
Coping through medication	0.022	0.34	0.13, 0.85
**9/11 database**			
*Survivors with postdisaster major depression at baseline*			
Coping through medication	0.046	0.13	0.02, 0.96

^a^ Independent covariates entered simultaneously in these models included baseline demographics (sex, age, racial minority, married status, college education), trauma exposure variables (injury in the disaster, and also for the 9/11 data: direct exposure, witnessed exposure, and indirect exposure), baseline diagnostic variables (predisaster major depression, disaster-related PTSD, postdisaster alcohol abuse/dependence), and baseline methods of coping with upset about the disaster (through family/friends, mental health treatment, drinking alcohol, and use of medication). Only variables significantly associated with the dependent variable are listed. SE = standard error; OR = odds ratio; CL = confidence limits.

**Table 2:** Multivariate models predicting current employment at follow up in two databases ^a^.

	β	SE	*p*	OR	95% CL
**10-disaster database**					
*Survivors who were employed at baseline* ^b^					
Age (years)	−0.10	0.02	<0.001		
**9/11 database**					
*Survivors who were employed at baseline* ^b^					
Age (years)	−0.22	0.09	0.015		
Coping through family and friends			0.013	75.06	2.57, >999.99
*Survivors who were employed and had postdisaster major depression at baseline* ^c^					
Age (years)	−0.24	0.10	0.012		
Indirect exposure			0.039	0.04	0.00, 0.83
Disaster-related PTSD			0.047	27.88	1.05, 743.28
Remission from depression			0.017	28.97	1.87, 448.98
Coping through family and friends			0.011	47.75	2.54, 896.38

^a^ Independent covariates entered simultaneously in these models included baseline demographics (sex, age, racial minority, married status, college education), trauma exposure variables (injury in the disaster, and also for the 9/11 data: direct exposure, witnessed exposure, and indirect exposure), baseline diagnostic variables (predisaster major depression, disaster-related PTSD, postdisaster alcohol abuse/dependence), and baseline methods of coping with upset about the disaster (through family/friends, mental health treatment, drinking alcohol, and use of medication). Only variables significantly associated with the dependent variable are listed. ^b^ Independent covariates also include postdisaster major depression. Employment was slightly greater (*p* < 0.05) for individuals whose last follow-up interview was at three years rather than one year; thus, this variable was included as an independent covariate in the model, but it was not significantly associated with the dependent variable in this model. ^c^ Independent covariates also include remission from postdisaster major depression. SE = standard error; OR = odds ratio; CL = confidence limits.

**Table 3:** Multivariate model predicting number of functioning problems at follow up in Oklahoma City bombing data ^a^.

	*p*	OR	95% CL
Baseline postdisaster alcohol abuse/dependence	0.046	0.16	0.00, 7.06
Injured in the disaster	0.038	0.87	0.03, 26.46

^a^ Independent covariates entered simultaneously in these models included baseline demographics (sex, age, racial minority, married status, college education), trauma exposure variables (injury in the disaster, and also for the 9/11 data: direct exposure, witnessed exposure, and indirect exposure), baseline diagnostic variables (predisaster major depression, disaster-related PTSD, postdisaster alcohol abuse/dependence), and baseline methods of coping with upset about the disaster (through family/friends, mental health treatment, drinking alcohol, and use of medication). Only variables significantly associated with the dependent variable are listed. SE = standard error; OR = odds ratio; CL = confidence limits.
